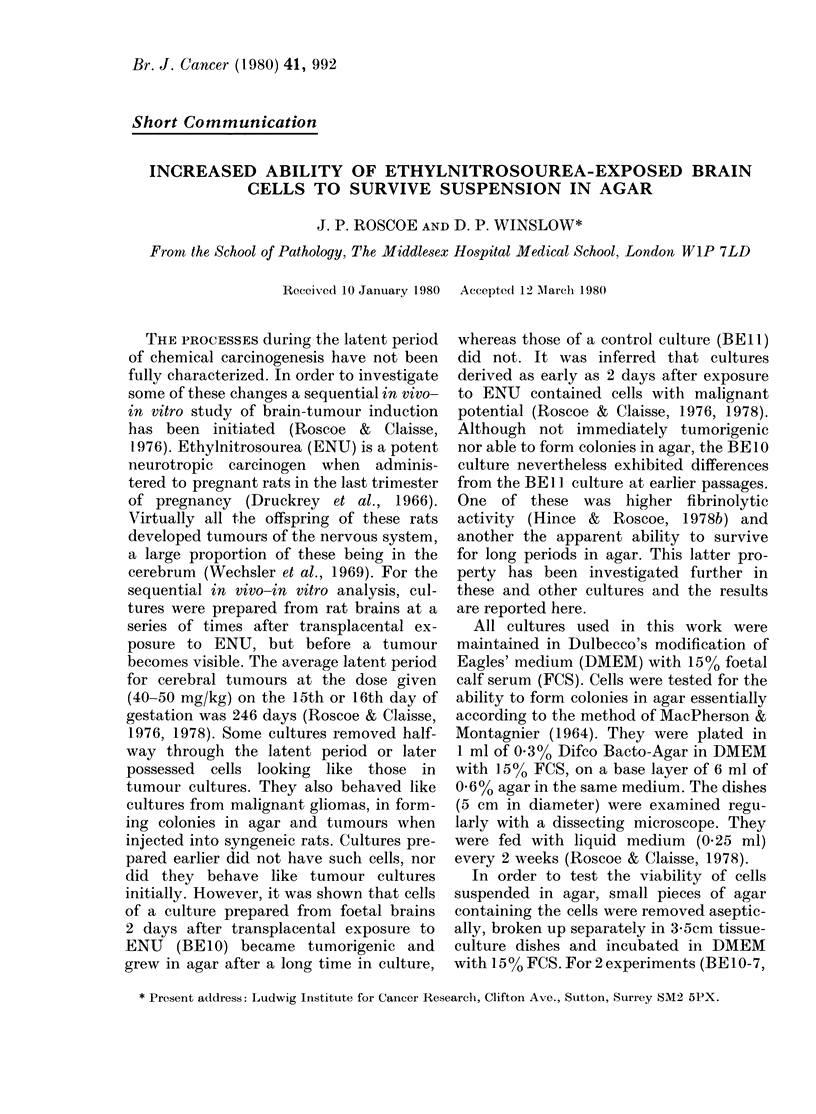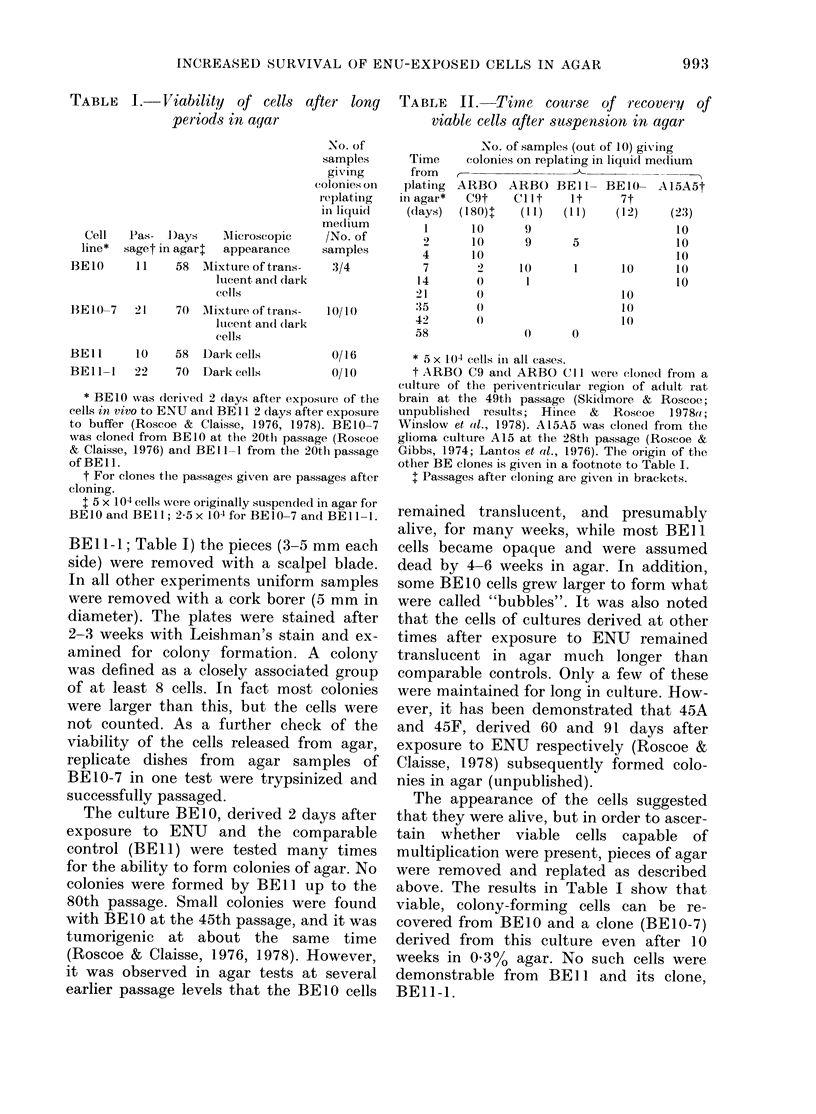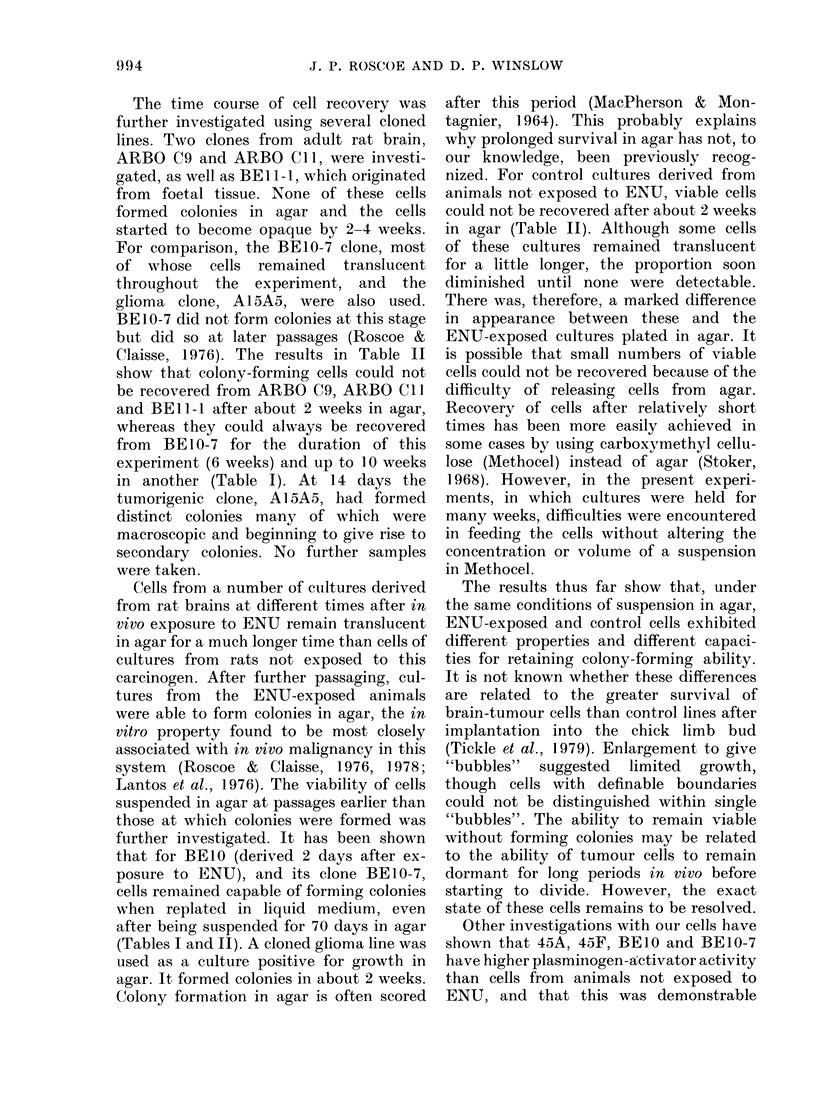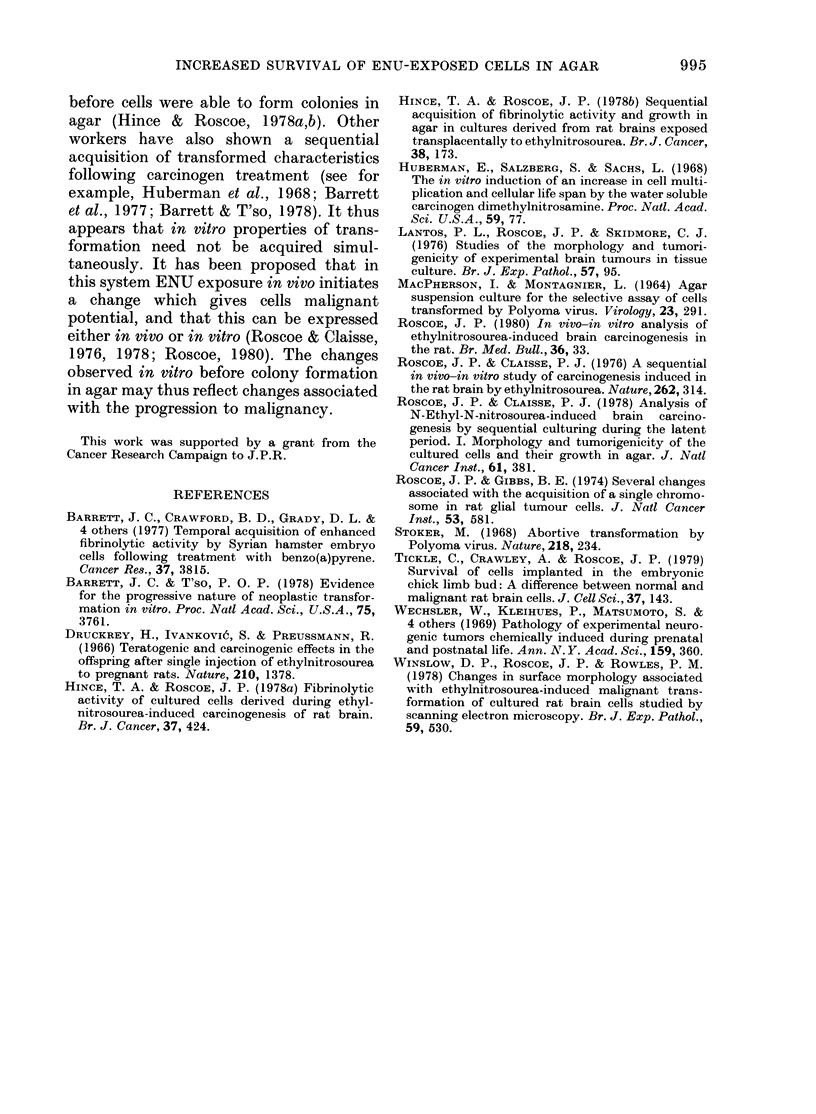# Increased ability of ethylnitrosourea-exposed brain cells to survive suspension in agar.

**DOI:** 10.1038/bjc.1980.180

**Published:** 1980-06

**Authors:** J. P. Roscoe, D. P. Winslow


Br. J. Cancer (1980) 41, 992

*Short Communication*

### INCREASED ABILITY OF ETHYLNITROSOUREA-EXPOSED BRAIN CELLS TO SURVIVE SUSPENSION IN AGAR

J. P. ROSCOE AND D. P. WINSLOW*

*From the School of Pathology, The Middlesex Hospital Medical School, London W1P 7LD*

Received 10 January 1980 Accepted 12 March 1980

THE PROCESSES during the latent period of chemical carcinogenesis have not been fully characterized. In order to investigate some of these changes a sequential *in vivo–in vitro* study of brain-tumour induction has been initiated (Roscoe & Claisse, 1976). Ethylnitrosourea (ENU) is a potent neurotropic carcinogen when administered to pregnant rats in the last trimester of pregnancy (Druckrey *et al.*, 1966). Virtually all the offspring of these rats developed tumours of the nervous system, a large proportion of these being in the cerebrum (Wechsler *et al.*, 1969). For the sequential *in vivo–in vitro* analysis, cultures were prepared from rat brains at a series of times after transplacental exposure to ENU, but before a tumour becomes visible. The average latent period for cerebral tumours at the dose given (40–50 mg/kg) on the 15th or 16th day of gestation was 246 days (Roscoe & Claisse, 1976, 1978). Some cultures removed halfway through the latent period or later possessed cells looking like those in tumour cultures. They also behaved like cultures from malignant gliomas, in forming colonies in agar and tumours when injected into syngeneic rats. Cultures prepared earlier did not have such cells, nor did they behave like tumour cultures initially. However, it was shown that cells of a culture prepared from foetal brains 2 days after transplacental exposure to ENU (BE10) became tumorigenic and grew in agar after a long time in culture, whereas those of a control culture (BE11) did not. It was inferred that cultures derived as early as 2 days after exposure to ENU contained cells with malignant potential (Roscoe & Claisse, 1976, 1978). Although not immediately tumorigenic nor able to form colonies in agar, the BE10 culture nevertheless exhibited differences from the BE11 culture at earlier passages. One of these was higher fibrinolytic activity (Hince & Roscoe, 1978*b*) and another the apparent ability to survive for long periods in agar. This latter property has been investigated further in these and other cultures and the results are reported here.

All cultures used in this work were maintained in Dulbecco's modification of Eagles' medium (DMEM) with 15% foetal calf serum (FCS). Cells were tested for the ability to form colonies in agar essentially according to the method of MacPherson & Montagnier (1964). They were plated in 1 ml of 0·3% Difco Bacto-Agar in DMEM with 15% FCS, on a base layer of 6 ml of 0·6% agar in the same medium. The dishes (5 cm in diameter) were examined regularly with a dissecting microscope. They were fed with liquid medium (0·25 ml) every 2 weeks (Roscoe & Claisse, 1978).

In order to test the viability of cells suspended in agar, small pieces of agar containing the cells were removed aseptically, broken up separately in 3·5cm tissue-culture dishes and incubated in DMEM with 15% FCS. For 2 experiments (BE10-7,

* Present address: Ludwig Institute for Cancer Research, Clifton Ave., Sutton, Surrey SM2 5PX.

TABLE I.—*Viability of cells after long periods in agar*

| Cell line* | Passage† | Days in agar‡ | Microscopic appearance | No. of samples giving colonies on replating in liquid medium /No. of samples |
|---|---|---|---|---|
| BE10 | 11 | 58 | Mixture of translucent and dark cells | 3/4 |
| BE10–7 | 21 | 70 | Mixture of translucent and dark cells | 10/10 |
| BE11 | 10 | 58 | Dark cells | 0/16 |
| BE11–1 | 22 | 70 | Dark cells | 0/10 |

* BE10 was derived 2 days after exposure of the cells *in vivo* to ENU and BE11 2 days after exposure to buffer (Roscoe & Claisse, 1976, 1978). BE10–7 was cloned from BE10 at the 20th passage (Roscoe & Claisse, 1976) and BE11–1 from the 20th passage of BE11.

† For clones the passages given are passages after cloning.

‡ $5 \times 10^4$ cells were originally suspended in agar for BE10 and BE11; $2{\cdot}5 \times 10^4$ for BE10–7 and BE11–1.

TABLE II.—*Time course of recovery of viable cells after suspension in agar*

| Time from plating in agar* (days) | No. of samples (out of 10) giving colonies on replating in liquid medium: ARBO C9† (180)‡ | ARBO C11† (11) | BE11–1† (11) | BE10–7† (12) | A15A5† (23) |
|---|---|---|---|---|---|
| 1 | 10 | 9 | | | 10 |
| 2 | 10 | 9 | 5 | | 10 |
| 4 | 10 | | | | 10 |
| 7 | 2 | 10 | 1 | 10 | 10 |
| 14 | 0 | 1 | | | 10 |
| 21 | 0 | | | 10 | |
| 35 | 0 | | | 10 | |
| 42 | 0 | | | 10 | |
| 58 | | 0 | 0 | | |

* $5 \times 10^4$ cells in all cases.

† ARBO C9 and ARBO C11 were cloned from a culture of the periventricular region of adult rat brain at the 49th passage (Skidmore & Roscoe; unpublished results; Hince & Roscoe 1978*a*; Winslow *et al.*, 1978). A15A5 was cloned from the glioma culture A15 at the 28th passage (Roscoe & Gibbs, 1974; Lantos *et al.*, 1976). The origin of the other BE clones is given in a footnote to Table I.

‡ Passages after cloning are given in brackets.

BE11-1; Table I) the pieces (3–5 mm each side) were removed with a scalpel blade. In all other experiments uniform samples were removed with a cork borer (5 mm in diameter). The plates were stained after 2–3 weeks with Leishman's stain and examined for colony formation. A colony was defined as a closely associated group of at least 8 cells. In fact most colonies were larger than this, but the cells were not counted. As a further check of the viability of the cells released from agar, replicate dishes from agar samples of BE10-7 in one test were trypsinized and successfully passaged.

The culture BE10, derived 2 days after exposure to ENU and the comparable control (BE11) were tested many times for the ability to form colonies of agar. No colonies were formed by BE11 up to the 80th passage. Small colonies were found with BE10 at the 45th passage, and it was tumorigenic at about the same time (Roscoe & Claisse, 1976, 1978). However, it was observed in agar tests at several earlier passage levels that the BE10 cells remained translucent, and presumably alive, for many weeks, while most BE11 cells became opaque and were assumed dead by 4–6 weeks in agar. In addition, some BE10 cells grew larger to form what were called "bubbles". It was also noted that the cells of cultures derived at other times after exposure to ENU remained translucent in agar much longer than comparable controls. Only a few of these were maintained for long in culture. However, it has been demonstrated that 45A and 45F, derived 60 and 91 days after exposure to ENU respectively (Roscoe & Claisse, 1978) subsequently formed colonies in agar (unpublished).

The appearance of the cells suggested that they were alive, but in order to ascertain whether viable cells capable of multiplication were present, pieces of agar were removed and replated as described above. The results in Table I show that viable, colony-forming cells can be recovered from BE10 and a clone (BE10-7) derived from this culture even after 10 weeks in 0·3% agar. No such cells were demonstrable from BE11 and its clone, BE11-1.

The time course of cell recovery was further investigated using several cloned lines. Two clones from adult rat brain, ARBO C9 and ARBO C11, were investigated, as well as BE11-1, which originated from foetal tissue. None of these cells formed colonies in agar and the cells started to become opaque by 2–4 weeks. For comparison, the BE10-7 clone, most of whose cells remained translucent throughout the experiment, and the glioma clone, A15A5, were also used. BE10-7 did not form colonies at this stage but did so at later passages (Roscoe & Claisse, 1976). The results in Table II show that colony-forming cells could not be recovered from ARBO C9, ARBO C11 and BE11-1 after about 2 weeks in agar, whereas they could always be recovered from BE10-7 for the duration of this experiment (6 weeks) and up to 10 weeks in another (Table I). At 14 days the tumorigenic clone, A15A5, had formed distinct colonies many of which were macroscopic and beginning to give rise to secondary colonies. No further samples were taken.

Cells from a number of cultures derived from rat brains at different times after *in vivo* exposure to ENU remain translucent in agar for a much longer time than cells of cultures from rats not exposed to this carcinogen. After further passaging, cultures from the ENU-exposed animals were able to form colonies in agar, the *in vitro* property found to be most closely associated with *in vivo* malignancy in this system (Roscoe & Claisse, 1976, 1978; Lantos *et al.*, 1976). The viability of cells suspended in agar at passages earlier than those at which colonies were formed was further investigated. It has been shown that for BE10 (derived 2 days after exposure to ENU), and its clone BE10-7, cells remained capable of forming colonies when replated in liquid medium, even after being suspended for 70 days in agar (Tables I and II). A cloned glioma line was used as a culture positive for growth in agar. It formed colonies in about 2 weeks. Colony formation in agar is often scored after this period (MacPherson & Montagnier, 1964). This probably explains why prolonged survival in agar has not, to our knowledge, been previously recognized. For control cultures derived from animals not exposed to ENU, viable cells could not be recovered after about 2 weeks in agar (Table II). Although some cells of these cultures remained translucent for a little longer, the proportion soon diminished until none were detectable. There was, therefore, a marked difference in appearance between these and the ENU-exposed cultures plated in agar. It is possible that small numbers of viable cells could not be recovered because of the difficulty of releasing cells from agar. Recovery of cells after relatively short times has been more easily achieved in some cases by using carboxymethyl cellulose (Methocel) instead of agar (Stoker, 1968). However, in the present experiments, in which cultures were held for many weeks, difficulties were encountered in feeding the cells without altering the concentration or volume of a suspension in Methocel.

The results thus far show that, under the same conditions of suspension in agar, ENU-exposed and control cells exhibited different properties and different capacities for retaining colony-forming ability. It is not known whether these differences are related to the greater survival of brain-tumour cells than control lines after implantation into the chick limb bud (Tickle *et al.*, 1979). Enlargement to give "bubbles" suggested limited growth, though cells with definable boundaries could not be distinguished within single "bubbles". The ability to remain viable without forming colonies may be related to the ability of tumour cells to remain dormant for long periods *in vivo* before starting to divide. However, the exact state of these cells remains to be resolved.

Other investigations with our cells have shown that 45A, 45F, BE10 and BE10-7 have higher plasminogen-activator activity than cells from animals not exposed to ENU, and that this was demonstrable before cells were able to form colonies in agar (Hince & Roscoe, 1978*a*,*b*). Other workers have also shown a sequential acquisition of transformed characteristics following carcinogen treatment (see for example, Huberman *et al.*, 1968; Barrett *et al.*, 1977; Barrett & T'so, 1978). It thus appears that *in vitro* properties of transformation need not be acquired simultaneously. It has been proposed that in this system ENU exposure *in vivo* initiates a change which gives cells malignant potential, and that this can be expressed either *in vivo* or *in vitro* (Roscoe & Claisse, 1976, 1978; Roscoe, 1980). The changes observed *in vitro* before colony formation in agar may thus reflect changes associated with the progression to malignancy.

This work was supported by a grant from the Cancer Research Campaign to J.P.R.

### REFERENCES

BARRETT, J. C., CRAWFORD, B. D., GRADY, D. L. & 4 others (1977) Temporal acquisition of enhanced fibrinolytic activity by Syrian hamster embryo cells following treatment with benzo(a)pyrene. *Cancer Res.*, **37**, 3815.

BARRETT, J. C. & T'SO, P. O. P. (1978) Evidence for the progressive nature of neoplastic transformation *in vitro*. *Proc. Natl Acad. Sci., U.S.A.*, **75**, 3761.

DRUCKREY, H., IVANKOVIĆ, S. & PREUSSMANN, R. (1966) Teratogenic and carcinogenic effects in the offspring after single injection of ethylnitrosourea to pregnant rats. *Nature*, **210**, 1378.

HINCE, T. A. & ROSCOE, J. P. (1978*a*) Fibrinolytic activity of cultured cells derived during ethylnitrosourea-induced carcinogenesis of rat brain. *Br. J. Cancer*, **37**, 424.

HINCE, T. A. & ROSCOE, J. P. (1978*b*) Sequential acquisition of fibrinolytic activity and growth in agar in cultures derived from rat brains exposed transplacentally to ethylnitrosourea. *Br. J. Cancer*, **38**, 173.

HUBERMAN, E., SALZBERG, S. & SACHS, L. (1968) The *in vitro* induction of an increase in cell multiplication and cellular life span by the water soluble carcinogen dimethylnitrosamine. *Proc. Natl. Acad. Sci. U.S.A.*, **59**, 77.

LANTOS, P. L., ROSCOE, J. P. & SKIDMORE, C. J. (1976) Studies of the morphology and tumorigenicity of experimental brain tumours in tissue culture. *Br. J. Exp. Pathol.*, **57**, 95.

MACPHERSON, I. & MONTAGNIER, L. (1964) Agar suspension culture for the selective assay of cells transformed by Polyoma virus. *Virology*, **23**, 291.

ROSCOE, J. P. (1980) *In vivo–in vitro* analysis of ethylnitrosourea-induced brain carcinogenesis in the rat. *Br. Med. Bull.*, **36**, 33.

ROSCOE, J. P. & CLAISSE, P. J. (1976) A sequential *in vivo–in vitro* study of carcinogenesis induced in the rat brain by ethylnitrosourea. *Nature*, **262**, 314.

ROSCOE, J. P. & CLAISSE, P. J. (1978) Analysis of N-Ethyl-N-nitrosourea-induced brain carcinogenesis by sequential culturing during the latent period. I. Morphology and tumorigenicity of the cultured cells and their growth in agar. *J. Natl Cancer Inst.*, **61**, 381.

ROSCOE, J. P. & GIBBS, B. E. (1974) Several changes associated with the acquisition of a single chromosome in rat glial tumour cells. *J. Natl Cancer Inst.*, **53**, 581.

STOKER, M. (1968) Abortive transformation by Polyoma virus. *Nature*, **218**, 234.

TICKLE, C., CRAWLEY, A. & ROSCOE, J. P. (1979) Survival of cells implanted in the embryonic chick limb bud: A difference between normal and malignant rat brain cells. *J. Cell Sci.*, **37**, 143.

WECHSLER, W., KLEIHUES, P., MATSUMOTO, S. & 4 others (1969) Pathology of experimental neurogenic tumors chemically induced during prenatal and postnatal life. *Ann. N.Y. Acad. Sci.*, **159**, 360.

WINSLOW, D. P., ROSCOE, J. P. & ROWLES, P. M. (1978) Changes in surface morphology associated with ethylnitrosourea-induced malignant transformation of cultured rat brain cells studied by scanning electron microscopy. *Br. J. Exp. Pathol.*, **59**, 530.